# The Psychometric Evaluation of the Connor-Davidson Resilience Scale Using a Chinese Military Sample

**DOI:** 10.1371/journal.pone.0148843

**Published:** 2016-02-09

**Authors:** Yuanjun Xie, Li Peng, Xin Zuo, Min Li

**Affiliations:** Department of military psychology, College of Psychology, Third Military Medical University, Chongqing, China; University of Stellenbosch, SOUTH AFRICA

## Abstract

This study examined the psychometric properties of the Connor-Davidson Resilience Scale (CD-RISC) with a Chinese military population with the aim of finding a suitable instrument to quantify resilience in Chinese military service members. The confirmatory factor analysis results did not support the factorial structure of the original or the Chinese community version of the CD-RISC, but the exploratory factor analysis results revealed a three-factor model (composed of Competency, Toughness, and Adaptability) that seemed to fit. Moreover, the repeat confirmatory factory analysis replicated the three-factor model. Additionally, the CD-RISC with a Chinese military sample exhibited appropriate psychometric properties, including internal consistency, test-retest reliability, and structural and concurrent validity. The revised CD-RISC with a Chinese military sample provides insight into the resilience measurement framework and could be a reliable and valid measurement for evaluating resilience in a Chinese military population.

## Introduction

Military operations always subject military service members to various types of stressors, such as training exercises, heavy workloads, and family separations [1, 2]. These stressors can lead to a variety of negative health consequences for some soldiers, including symptoms of major depression, anxiety, and posttraumatic stress disorder [3] and suicidal behaviors [4]. Although many people experience negative physical and mental health effects following exposure to adversity, many others show remarkable resilience to experiencing stress [5]. Therefore, resilience is currently receiving increasing attention in policy and practice [6, 7] because of its potential influence on health, well-being and quality of life. Resilience is considered a highly influential factor that protects individuals from the negative impact of stress [8] and increases the ability to deal with considerable challenges [9] and to recover rapidly from negative emotional experiences [10].

Accordingly, military organizations have developed an array of resiliency-building training programs to equip military personnel to survive and thrive when facing extreme situations. One example is the comprehensive soldier fitness program (CSF), an integrated and proactive approach launched by the United States Army to promote resilience within the army community [7]. Recently, some Chinese military psychology researchers have initiated adaptive resilience training programs to enhance resilience among Chinese military personnel [11].

Evaluations of the interventions and programs designed to enhance resilience require measurement tools with adequate evidence of reliability and validity. Nevertheless, the varying definitions and the construct of resilience have created considerable challenges for measuring resilience [12]. Herein, resilience is defined as personality traits that reflect the capability to cope successfully and recover from substantial adversity [13]. Within this conceptual framework, a dozen psychological instruments have been developed to assess resilience [14].

Among these instruments, a newly developed scale—the Connor-Davidson Resilience Scale (CD-RISC) [15], a clinical assessment of the ability to cope with stress—has been broadly applied in various countries [11, 16–19]. This scale consists of five factors and 25 self-reported items. The factors are as follows: personal competency, high standards, and tenacity (8 items), trust or tolerance of negative affect and stress (7 items), acceptance of change and secure relationships (5 items), control (3 items) and spirituality (2 items). However, the scale’s original factorial framework lacked of robust replication [16, 20]. Some reasons have presented possible explanations for this limitation. For example, there were some measurement methodology drawbacks to the design and development of the scale. First, the item distribution of the fourth and fifth factors (with 3 items and 2 items, respectively) seemed to be questionable because fewer items were insufficient to form a stable factor [21]. Second, the use of the Kaiser-Guttmann criterion alone to decide how many factors to retain was a poor choice because it can easily lead to overfactoring [22]. Finally, the names of the first three factors could be confusing because they combined dissimilar concepts [23].

Another important explanation for the instability of the factor structure was the diversity of resilience understanding in different cultural settings [24]. Because adversity and hardship may have different meanings for people with different cultural backgrounds, the meaning and framework of resilience could not be equal across cultures [20]. Particularly, the fifth factor of the original version (spiritual descriptions, such as sometimes fate or God can help) may be misunderstood among those without religious beliefs who live in non-western countries. To illustrate, spiritual influence did not emerge as independent factor in the translated CD-RISC with a Chinese community sample [20], which instead yielded a 3-factor structure of resilience, labeled Tenacity, Strength, and Optimism. This finding suggested that resilience measurements from the West can be helpful and suitable for understanding Chinese adaptive behaviors, but their structure may require some modification to accommodate Chinese culture [20].

Taking into account the difficulty of establishing a clear construct for the CD-RISC, this scale needs to be revised to meet the specific research area. Therefore, the aim of this study was to investigate the previous models of the CD-RISC to determine whether they match the data from a Chinese military sample and to examine the reliability and validity of the CD-RISC for the current sample. Our study primarily focused on the original version of the CD-RISC [15] and the CD-RISC with a Chinese community version [20] because the former has been broadly applied to the different populations and the latter has been properly translated into Mandarin.

Additionally, in the present study, the variables of self-esteem and positive and negative affect were considered concurrent validity indicators of the CD-RISC because the self-esteem and positive and negative affect scales have been properly translated and are widely used in mainland China and because these two variables have consistently been highly correlated with resilience in previous studies [19, 25, 26].

## Method

### Participants and procedure

The participants were selected from four military units—the army (40%), the marines (38%), and Special Forces (22%)—using convenience sampling and were divided into two groups. In group 1, 1,573 valid participants were aged 18 to 41 years (M = 22.27, SD = 4.57), and 77.9% were male; in group 2, 784 valid participants were aged 18 to 44 years (M = 23.75, SD = 5.31), and 87.0% were male. Following a brief introduction to the study, the researchers administered the paper-and-pencil questionnaires to the participants in small groups. The study was approved by the Third Military Medical University Ethic Committee, and all participants provided written informed consent.

### Measures

#### Chinese community version of the Connor-Davidson Resilience Scale

The Chinese community version of the CD-RISC comprised 3 factors and 25 items [20] and showed good reliability and validity. The internal consistency values of the three factors were 0.88, 0.80 and 0.60, respectively. The items were rated on a 5-point Likert scale from 0 (not true at all) to 4 (true nearly all the time).

#### Rosenberg self-esteem scale

This 10-item scale is widely used to evaluate an individual’s general sense of self-worth and self-acceptance [27]. The Cronbach alpha values of the scale were 0.88 for the English version [28] and 0.84 for the Chinese version [29]. The items used a 4-point Likert scale ranging from 1 (strongly disagree) to 4 (strongly agree).

#### Positive and negative affect scale

The original 20-item scale was split into two independent subscales: positive affect and negative affect [30]. Each subscale has ten affective descriptors that are rated on a 5-point Likert scale ranging from 1 (very slightly or not at all) to 5 (extremely). The reported Cronbach’s alpha values of the two subscales in the original version were 0.88 and 0.87; for the adapted Chinese version, they were 0.85 and 0.84 [31].

### Data analysis

The first confirmatory factor analysis (CFA) was performed to examine the previous factor models from the CD-RISC and to determine which model would be supported. Moreover, parallel analysis (PA) and exploratory factor analysis (EFA) were used to determine the possible factor structure. The second CFA was used to verify the factor solution extracted from the EFA. Considering the data categorization based on the Likert response scales, the CFA was performed with MPLUS 7.3[32] using a polychoric correlation matrix. The fit indices had the following indicators: χ^2^/df (the ratio of the chi-square to the degree of freedom), the comparative fit index (CFI), the Tucker-Lewis index (TLI), the root-mean-square error of approximation (RMSEA), and the weighted root mean square residual (WRMR). The acceptability criteria for these indicators were as follows: χ^2^/df less than 5 [33], both CFI and TLI greater than 0.90 [34], RMSEA smaller than 0.08 [35], and WRMR no more than 1 [36].

## Results

### The CFA of the different models of the CD-RISC

The CFA was used with the weighted least squares means and variance (WLSMV) estimator to replicate the previous factor models, including the original version and the CD-RISC with a Chinese community sample, based on the data from group 1. The details of these models’ fit are shown in the Table 1. The fit indices of the models failed to meet the standard values, and the results suggest that these models might not be supported by the present sample.

**Table 1:** Goodness-of-fit for the CD-RISC with different models.

Different Versions of CD-RISC	X^2^/df	CFI	TLI	RMSEA	WRMR	AIC
Five factors of original model	11.64	0.89	0.89	0.08	2.28	2111.63
Three factors of Chinese community model	12.07	0.88	0.87	0.08	2.37	2263.49
The present study model	4.98	0.95	0.94	0.07	1.25	632.02

### The EFA of the CD-RISC with a Chinese military sample

Because the factorial structures of previous studies were not confirmed with CFA, the next step was to investigate the possible factor solution of the CD-RISC by the EFA using MPLUS with polychoric matrix. First, the PA program was used to determine the number of extracted factors [37]. The PA result indicated that the first three actual eigenvalues were greater than their corresponding random 95th percentile eigenvalues for the data from group 1. Likewise, the scree plot showed that the number of factors retained seemed appropriate based on the cut-off of the step decline.

Therefore, next, the number of extracted factors was set at three, and the EFA was run using MPLUS with the WLSMV estimator and oblique rotation based on group 1. The results showed that there were three eigenvalues greater than 1 and that the fit values of the EFA model (CFI = 0.96, TLI = 0.9, and RMSEA = 0.06) were acceptable. The item weight and factor selection details are shown in Table 2. In particular, the factor loadings of item 3 (sometimes fate and God can help), item 15 (prefer to take the lead in problem solving), item 18 (make unpopular or difficult decisions), and item 20 (have to act on a hunch) were less than 0.40, which did not meet the lowest acceptable threshold [38]. Moreover, these items did not load to any factor. The subsequent analysis did not include these items.

**Table 2:** The eigenvalues and factor pattern of the CD-RISC with Chinese military sample.

Item numbers		Factor loadings		
		I	II	III
11	One can achieve one's goals	0.78		
5	Past success gives confidence for new challenge	0.74		
10	Best effort no matter what	0.72		
16	Not easily discouraged by failure	0.72		
6	See the humorous side of things	0.71		
17	Think of self as strong person	0.70		
14	Under pressure, focus and think clearly	0.69		
7	Coping with stress strengthens	0.54		
9	Things happen for a reason	0.47		
24	One works to attain one's goals		0.90	
23	I like challenge		0.80	
21	Strong sense of purpose		0.72	
12	When things look hopeless, I don't give up		0.69	
22	In control of my life		0.64	
8	Tend to bounce back after illness or hardship		0.54	
4	Can deal with whatever comes			0.69
13	Know where to get help			0.69
1	Able to adapt to change			0.61
19	Can handle unpleasant feelings			0.61
25	Pride in your achievements			0.58
2	Close and secure relationships			0.46
Eigenvalues		9.95	1.69	1.45

*Notes*: Factor loading with absolute value below 0.40 did not displayed in the table. EFA was performed with oblique rotation and WLSMV estimation method.

The first factor was labeled Competency. It comprised 9 items that described personal characteristics, such as confidence, optimism, virtue and self-efficacy, that could be considered protective factors that contribute to resilience and buffer the negative effects of adversity and trauma. The second factor, comprising 6 items, was labeled Toughness and referred to the ability to resist or bounce back from stress, which conforms to the nature of resilience. The last factor that was primarily loaded by 6 items was labeled Adaptability and focused on an individual’s adaptation to change, emotional regulation, and use of resources, measuring one’s adaptive capacity in disadvantageous situations.

### Validity of the CD-RISC with a Chinese military sample

The second CFA was used to replicate the 3-factor model extracted from the EFA of the data from group 2. The fit indices are displayed in Table 1. Although the WRMR value was slightly greater than 1, the overall fit indices of the model were acceptable, and the Akaike information criterion (AIC) values of the model comparison indicator showed that this model was better than other two. The results suggested that the 3-factor model from the Chinese military sample seemed to be reasonable and replicable.

In addition, the correlation matrix is listed in the Table 3. The total score of the revised CD-RISC was positively correlated with self-esteem (r = 0.27, P<0.01) and positive affect (r = 0.52, P<0.01) and inversely related to negative affect (r = -0.26, P<0.01). The results indicated that the concurrent validity of the revised CD-RISC with related variables was satisfied in the present sample.

**Table 3:** Ordinal reliability coefficients, correlation coefficients and concurrent validity evidence on the CD-RISC with Chinese military sample.

	Ordinal alpha	Factor 1	Factor 2	Factor 3	Self-esteem	PA	NA
CD-RISC	0.94	0.94	0.89	0.88	0.27	0.52	-0.26
Factor 1	0.89		0.76	0.77	0.25	0.46	-0.24
Factor 2	0.86			0.68	0.20	0.47	-0.24
Factor 3	0.81				0.27	0.48	-0.21
*M*		24.6	19.2	13.1	28.1	28.6	20.0
*SD*		3.1	4.6	3.3	2.1	6.1	6.6

*Note*: Factor 1: Competency, Factor 2: Toughness, Factor 3: Adaptability. PA = positive affect, NA = negative affect. Correlation coefficients were significant at the alpha 0.01 level.

### Reliability of the CD-RISC with a Chinese military sample

The ordinal reliability coefficients were calculated using the Psych package of R (www.r-project.org) with a polychoric correlation matrix, as recommended by Zumbo and colleague [39], because ordinal alphas more accurately estimate reliability than Cronbach’s alphas when the data is derived from Likert-type items [40]. The results of the calculation are presented in Table 3. The ordinal alpha values obtained ranged from 0.81 to 0.94. The correlation coefficients between the total scale scores and the subscale scores were moderate to high (Table 3). The test-retest reliability (P<0.001) across an interval of two months (for 319 participants from group 1) was 0.66.

## Discussion

The results based on factor analysis suggest that a three-factor structure of the CD-RISC with a Chinese military sample was superior to the original five-factor pattern. The present three-factor solution comprising Competency (nine items), Toughness (6 items), and Adaptability (6 items) emerged as the best-fitting model. When tested, the three-factor model displayed favorable internal consistency, consistent structure validity and good concurrent validity.

Consistent with the majority of the other research examining the factor structure of this scale, the present study found no evidence to support the original five-factor model. Comparisons of the results of the present three-factor model with the results of previous studies suggest that differences in background characteristics, such as cultural factors, may have contributed to some of the observed differences in factorial construction because cultural contexts influence the awareness of resilience [24] and the meaning and structure of resilience differ among diverse cultures [41]. In particular, all religious beliefs and activities are banned by the Chinese military, and so the original spiritual items, such as sometimes fate or God can help, might be confusing to Chinese military personnel. Moreover, spirituality is not a significant predictor of resilience [42]; rather, it is a means of coping. In addition, other items from the original version, such as items 15, 18, and 20, could not apply in the military culture because service members must obey orders and commands rather making decisions or taking action independently.

Furthermore, an understanding of the nature of resilience requires a clear definition of the meaning and structure of resilience. This scale, which measures resilience at the individual level, would have benefited from greater theoretical clarification [43]. A meta-model of stress, emotion, and performance offers new insights into the role of resilience in the stress process [44]. Ongoing stress processes are moderated by resilience-related variables, including hardiness, self-esteem, and positive affect; consequently, these variables influence the stress process at multiple stages, such as the appraisal of stressors and the selection of coping strategies.

This means that the personal characteristics-related attributes such as self-esteem [45], self-confidence [46], optimism [15], patience [47], hardiness [48], positive emotions [49], and self-efficacy [50] can be considered personal resources or protective factors for resilience. Resilience-related protective factors protect people against the psychological risks associated with adversity [51]. Competence (factor 1) represents a resource caravan or an aggregation of personal qualities that help to prevent maladjustment resulting from stress and adversity. Therefore, Competence reflects an integral feature of resilience because resilience is a trait that one either has or does not have [12]. Individuals with resilient qualities can effectively address internal and external stressors and maintain biopsychospiritual homeostasis [52].

Toughness (factor 2) is the ability to withstand adverse situations or recover from negative experiences, which is consistent with the original and basic meaning of resilience (bouncing back or returning to normal levels of function). The inclusion of Toughness means that resilience is not only about overcoming a stressful situation but also helps to make those who rebound from adversity stronger and more resourceful. Hence, a Chinese soldier with resilience consciously integrates control, goal-setting, perseverance and recovery when he or she is encounters a frustrating situation. Accordingly, Toughness is fundamentally important for surviving or dealing with challenges and demands [53].

Adaptability factor (factor 3) refers to the ability to produce an effective response in a changing situation [54]. It is most important for military personnel because adaptability is an integral component of military culture and is heavily emphasized in tasks and training [55]. The pace of military operations requires military service members to take effective actions under changing and extreme conditions. In addition, positive adaptation involves finding and using the appropriate resource to address the difficult situation and effectively regulate emotions. Consequently, adaptability is one of the primary attributes of resilience [56].

A generic model of resilience in response to trauma identifies some key variables in the determination of resilience behavior [57]: personality, affect modulation, ego defense, coping style, and the mobilization and utilization of protective factors. These variables can work together to produce a continuum of adaptive and resilience behaviors in the wake of adversity. Thus, the structure of resilience emerged as a triad of competency, toughness, and adaptability, which could be identified as the key characteristics of resilience that contributing to effective coping and adaptation. Therefore, the present 3-factor construct of resilience may reflect the essential nature of resilience and provide an insight into the resilience measurement framework.

In addition, the methodology used in the present study enhanced the reliability of the results. For example, the use of many methods to determine the number of retained factors was more accurate than the use of a single method. Data analysis using a polychoric matrix was suitable to the data, which was drawn from categorical variables. Moreover, the item distribution of 9 items, 6 items and 6 items corresponding to the 3 factors of the present model appeared to be more reasonable than the item distribution of the original version. The reliability coefficients of the CD-RISC with a Chinese military sample (ordinal alpha = 0.94) were higher than those of the original scale (Cronbach’s alpha = 0.89) and the Chinese community version (Cronbach’s alpha = 0.91).

In conclusion, the present study provided preliminary information on the CD-RISC with a Chinese military sample. This initial attempt suggests that the construct of resilience was reasonable. The revised CD-RISC was a reliable and valid instrument for measuring resilience in a Chinese military population. Future research needs to focus on the validation of the revised CD-RISC and should continue to evaluate constructs related to resilience and on ways to build resilience.
